# Integrative Proteomic and Phosphoproteomic Analyses of Granulosa Cells During Follicular Atresia in Porcine

**DOI:** 10.3389/fcell.2020.624985

**Published:** 2021-01-15

**Authors:** Feng Yang, Qiang Liu, Yanhong Chen, Huizhen Ye, Han Wang, Shenming Zeng

**Affiliations:** ^1^National Engineering Laboratory for Animal Breeding, Key Laboratory of Animal Genetics and Breeding of the Ministry of Agriculture, College of Animal Science and Technology, China Agricultural University, Beijing, China; ^2^Beijing Advanced Innovation Center for Genomics, Center for Reproductive Medicine, Department of Obstetrics and Gynecology, Peking University Third Hospital, Beijing, China; ^3^Key Laboratory of Assisted Reproduction, Ministry of Education, Beijing, China; ^4^Beijing Key Laboratory of Reproductive Endocrinology and Assisted Reproductive Technology, Beijing, China

**Keywords:** porcine, follicular atresia, granulosa cell, proteomic, phosphoproteomic

## Abstract

Ovarian follicular atresia is a natural physiological process; however, the mechanism is not fully understood. In this study, quantitative proteomic and phosphoproteomic analyses of granulosa cells (GCs) in healthy (H), slightly atretic (SA), and atretic follicles (A) of porcine were performed by TMT labeling, enrichment of phosphopeptides, and liquid chromatography with tandem mass spectrometry (LC–MS/MS) analysis. In total, 6,201 proteins were quantified, and 4,723 phosphorylation sites of 1,760 proteins were quantified. In total, 24 (11 up, 13 down) and 50 (29 up, 21 down) proteins with a fold change (FC) > 5 were identified in H/SA and H/A, respectively. In addition, there were 20 (H/SA, up) and 39 (H/A, up) phosphosites with an FC > 7 that could serve as potential biomarkers for distinguishing different quality categories of follicles. Western blotting and immunofluorescence confirmed the reliability of the proteomic analysis. Some key proteins (e.g., MIF, beta catenin, integrin β2), phosphosites (e.g., S76 of caspase6, S22 and S636 of lamin A/C), pathways (e.g., apoptosis, regulation of actin cytoskeleton pathway), transcription factors (e.g., STAT5A, FOXO1, and BCLAF1), and kinases (e.g., PBK, CDK5, CDK12, and AKT3) involved in the atresia process were revealed *via* further analysis of the differentially expressed proteins (DEPs) and phosphorylated proteins (DEPPs). Further study showed that mutant caspase6 Ser76 to Ala increased the ratios of cleaved caspase6/caspase6 and cleaved caspase3/caspase3 and dephosphorylation of caspase6 at Ser76 increased cell apoptotic rate, a new potential pathway of follicular atresia. Collectively, the proteomic and phosphoproteomic profiling and functional research in the current study comprehensively analyzed the dynamic changes in protein expression and phosphorylation during follicular atresia and provided some new explanations regarding the regulation of this process.

## Introduction

In female mammals, less than 1% of follicles are selected, and the remainder are eliminated (Himelstein-Braw et al., 1976; Hurwitz and Adashi, 1992). Most follicles undergo a degenerative process known as atresia (Manabe et al., 2004). Follicular atresia was described as being common among all vertebrate groups in the early 1947 (Albert, 1947; Rastogi, 2010). In humans, the total germ cell number reaches a peak of 6.8 million at 5 months of gestational age. By the time of birth, this number has declined to 2 million, and only 300–400 thousand follicles are present at the onset of puberty (Baker, 1963; Hsueh et al., 1994). A woman usually ovulates only approximately 400 follicles during her reproductive life (Hsueh et al., 1994); therefore, more than 99.9% of human follicles undergo atresia. In porcine, the reserve of primordial follicles is estimated to be 10 million in the ovaries 10 days after birth, and the recruitment of resting primordial follicles into the growing pool begins during the fetal life (Manabe et al., 2004). However, at most, 1,600 oocytes will ovulate during the fertile life in porcine, and the others will disappear (Manabe et al., 2004). Atresia occurs continually throughout a female’s life, and follicular atresia is similar in pigs and humans. Pigs are economically important and have also been increasingly used as an alternative model for studying human health and disease and for testing new surgical and pharmacological treatments (Freeman et al., 2012). In addition, pigs are closely related to humans in terms of anatomy, genetics, and physiology (Meurens et al., 2012). Researchers often use porcine follicles to study follicular development and atresia, because there are various grades of follicles on their ovaries, which are easy to observe and manipulate.

It is widely accepted that ovarian follicular atresia is mediated primarily by granulosa cell (GC) apoptosis (Tilly et al., 1991; Monniaux et al., 1998; Yu et al., 2004; Logothetopoulos et al., 2010), which is regulated by internal and external factors (Hsueh et al., 1994). The GCs first undergo apoptosis, then the GC layer detaches from the follicular basement membrane (BM), after which, fragmentation of the BM begins, and finally, the follicle disappears (Manabe et al., 2004). Apoptosis of GCs is similar to other cell types, which exhibit cell membrane blebbing, DNA degradation, and protease activation; however, there are some specific characteristics of GC apoptosis. For example, during the initial steps of apoptosis, steroidogenesis is increased due to aggregation of the steroidogenic organelles in the perinuclear region and their exclusion from the apoptotic blebs. Actin cytoskeleton reorganization plays an important role in this compartmentalization, as well as in transmitting survival factors exerted by the BM (Amsterdam et al., 1998). Atresia is triggered when some essential factors supporting follicular development are lacking, in particular, the gonadotropins [follicle-stimulating hormone (FSH) and luteinizing hormone (LH)], growth factors, cytokines, steroids, and constituents of the extracellular matrix (ECM) in antral follicles (Monniaux et al., 1998). The Bcl-2 protein family plays an irreplaceable role during apoptosis. Recent results from experiments done by our group demonstrated that heat stress promotes BimEL phosphorylation at Thr112 through the JNK pathway and decreases the level of aromatase in porcine GC, thus damaging follicular development (Wu et al., 2019; Yang et al., 2020a). Atresia also involves active cellular processes including macrophage infiltration, phagocytosis, migration of fibroblasts from the theca, and the production of collagen (Bansal and Uppal, 2014). Gene expression level of an oocyte-secreted factor, *BMP15*, was upregulated three times in healthy follicles than in atretic follicles, whereas GDF9 was relatively unchanged (Hatzirodos et al., 2014). *INHA* and *INHBA*, which encode activins and inhibin, and *FST*, which produces follistatin, were all downregulated in bovine atretic follicles (Hatzirodos et al., 2014).

To study the dynamic changes in gene expression during the process of follicular atresia, transcriptome analysis was performed in porcine GCs from healthy and atresia follicles by two independent laboratories (Terenina et al., 2016; Zhang et al., 2018a). Some markers of follicular atresia, key genes, and pathways were found; however, the ultimate function executor in biological processes is proteins. The continuous advancement of proteomics technology provides the opportunity to gain a deeper understanding of the dynamic expression profiles of proteins during atresia. Protein phosphorylation is one of the most important posttranslational modifications (PTM) in living cells and is involved in various biological processes, including follicular atresia (Wu et al., 2019; Yang et al., 2020a). However, the dynamic spectrum of proteins and protein phosphorylation during the process of follicular atresia has not yet been elucidated. In addition, dynamics of transcription factors (TFs) and kinases during this process are not yet known. In this study, quantitative proteomic and phosphoproteomic analyses of the GCs of healthy (H), slightly atretic (SA), and atretic (A) porcine follicles were performed to further investigate the mechanisms of follicular atresia.

## Materials and Methods

### Classification of Healthy, Slightly Atretic, and Atretic Follicles and Recovery of GCs

Ovaries from commercial large white gilts aged about 5 months were collected at a local abattoir and transported to the laboratory in a vacuum flask containing sterile physiological saline (30–35°C) within 2 h of collection. Ovaries were washed twice with sterile physiological saline (37°C) containing 100 IU/L penicillin and 50 mg/L streptomycin. H, SA, and A follicles in porcine were classified according to previously established morphological criteria (Wang et al., 2012; Cao et al., 2015; Terenina et al., 2016). Briefly, healthy follicles were defined as vascularized theca internal and clear amber follicular fluid with no debris. The follicles lacking any of these criteria were classified as atretic. The slightly atretic and atretic follicles had gray theca internal and flocculent follicular fluid in varying degrees. Follicular (3–5 mm in diameter) contents from 10 ovaries were punctured by hypodermic needle, and cumulus-oocyte complex and ovarian tissue were discarded under a stereo microscope. The GCs were subsequently harvested by centrifuging. Samples were collected three times at different days.

### Protein Extraction, Concentration Measurement, and Trypsin Digestion

Protein extraction, concentration measurement, and trypsin digestion were done as previously described (Chen et al., 2015). Cells were lysed in lysis buffer containing 8 M urea, 10 mM dithiothreitol (DTT), 1% Protease Inhibitor Cocktail, and phosphatase inhibitors. Samples were centrifuged to remove debris, and the supernatant was collected. Finally, the protein was precipitated with cold 15% TCA for 2 h at 4°C. After centrifugation at 4°C at 5,000 × *g* for 10 min, the supernatant was discarded. The remaining precipitate was washed three times with cold acetone. The protein was then dissolved in buffer [8 M urea, 100 mM triethylammonium bicarbonate (TEAB), pH 8.0], and the protein concentration was determined using a 2-D Quant kit (GE Healthcare, Piscataway, NJ, United States) according to the manufacturer’s instructions. For digestion, the protein solution was reduced with 10 mM DTT for 1 h at 37°C and alkylated with 20 mM iodoacetamide (IAA) for 45 min in the dark at room temperature. For trypsin digestion, the protein sample was diluted by adding 100 mM TEAB to urea concentration less than 2 M. Finally, trypsin was added at a 1:50 trypsin-to-protein mass ratio for the first overnight digestion and at a 1:100 trypsin-to-protein mass ratio for the second 4 h digestion.

### TMT Labeling, HPLC Fractionation, and Affinity Enrichment of Phosphopeptides

After trypsin digestion, peptide was desalted by Strata X C18 SPE column (Phenomenex, CA, United States) and vacuum-dried. Peptide was reconstituted in 1 M TEAB and processed using a 6-plex TMT kit according to the manufacturer’s protocol. Briefly, one unit of TMT reagent (defined as the amount of reagent required to label 100 μg of protein) was thawed and reconstituted in 24 μl acetonitrile (ACN). The peptide mixtures were then incubated for 2 h at room temperature, pooled, desalted, and dried by vacuum centrifugation.

High-performance liquid chromatography (HPLC) fractionation and affinity enrichment of phosphopeptides were done as previously described (Chen et al., 2015). Briefly, the peptides were combined into eight fractions and dried by vacuum centrifuging. The phosphopeptides were enriched using immobilized metal affinity chromatography (IMAC). The supernatant containing phosphopeptides was collected and lyophilized for liquid chromatography with tandem mass spectrometry (LC–MS/MS) analysis.

### LC–MS/MS Analysis

Liquid chromatography with tandem mass spectrometryLC–MS/MS analysis was performed as previously described (Chen et al., 2015; Yuan et al., 2019). Briefly, after the peptides were dissolved using 0.1% formic acid (FA), samples were separated using an EASY-nLC 1000 ultrahigh performance liquid phase system and then subjected to NSI source, followed by Q-Exactive Plus system analysis. Peptides were selected and fragmented with a normalized collision energy of 30 eV fragment ions detected in the Orbitrap at a resolution of 17,500. For the proteomic, the gradient was composed of an increase from 7 to 25% solvent B (0.1% FA in 98% ACN) for 24 min, 25 to 40% for 8 min, and climbing to 80% in 4 min then holding at 80% for the last 4 min, all at a constant flow rate of 350 nl/min on an EASY-nLC 1000 UPLC system. For the phosphorproteomic, the gradient was composed of an increase from 4 to 22% solvent B (0.1% FA in 98% ACN) for 40 min, 22 to 35% for 12 min, and climbing to 80% in 4 min then holding at 80% for the last 4 min, all at a constant flow rate of 400 nl/min on an EASY-nLC 1000 UPLC system.

### Proteomic and Phosphorproteomic Database Search

Raw data were processed with Max MaxQuant search engine (v.1.5.2.8). Tandem mass spectra were searched against uniprot *Sus scrofa* (26,201 sequences) database. For the proteomics, Trypsin/P was specified as the cleavage enzyme allowing up to two missing cleavages. Mass error was set to 20 ppm for precursor ions and 0.02 Da for fragment ions. Carbamidomethyl on Cys was specified as fixed modification, and oxidation on Met and acetylation on protein N-term were specified as variable modifications. For the protein quantification method, TMT 6-plex was selected in MaxQuant. The false discovery rate (FDR) was adjusted to <1% at the protein and peptide spectrum match (PSM) levels. For the phosphoproteomics, Trypsin/P was specified as the cleavage enzyme allowing up to two missing cleavages. The maximum number of modifications per peptide was set to 5. Mass error was set to 20 ppm for precursor ions and 0.02 Da for fragment ions. Carbamidomethylation on Cys was specified as fixed modification, and oxidation on Met; phosphorylation on Ser, Thr, and Tyr; and acetylation on protein N-terminal were specified as variable modifications. FDR threshold for protein, peptide, and modification site was specified at 1%. Minimum peptide length was set at 7. For the quantification method, TMT-6-plex was selected. The site localization probability was set as >0.75. The mass spectrometry proteomics data have been deposited to the ProteomeXchange Consortium *via* the PRIDE partner repository with the dataset identifier PXD020899.

### GO, Protein Domain, KEGG Pathway Annotation, and Subcellular Localization

UniProt-GOA database was used to annotate protein Gene Ontology (GO) terms. Protein domain was annotated by InterProScan based on the protein sequence alignment method, and the InterPro domain database was used. Kyoto Encyclopedia of Genes and Genomes (KEGG) database was used to annotate the protein pathway. We used WoLF PSORT, a subcellular localization predication soft to predict subcellular localization.

### Motif Analysis

Soft motif-x was used to analyze the model of sequences constituted with amino acids in specific positions of modify-13-mers (6 amino acids upstream and downstream of the site) in all protein sequences. And, all the database protein sequences were used as background database parameter and other parameters with default.

### Protein Functional Enrichment

For each protein functional term, a two-tailed Fisher’s exact test was employed to test the enrichment of the differentially expressed protein (DEP) against all identified proteins. A term with a corrected *P* < 0.05 was considered significant.

### Functional Enrichment-Based Clustering

A *P* value of functional enrichment was collated from compare groups, retaining functional term that was at least enriched in one of the compare group with *P* < 0.05. Then, a *P* value matrix was transformed by -log10 and z-score for each functional category. These z scores were clustered by one-way hierarchical clustering (Euclidean distance, average linkage clustering). Cluster membership was visualized by a heat map using the “heatmap.2” function from the “gplots” R-package.

### TF Searching

The 1,490 known porcine TFs from the Animal Transcription Factor Database (AnimalTFDB3^1^) were used to search and analyze the expression patterns of the TFs in the proteome and phosphoproteome (Hu et al., 2019).

### Kinase Analysis

GPS 5.0 software was used for predicting kinase–substrate regulations. The corresponding kinase proteins in the kinase family were obtained by comparison with the kinase sequence in the IEKPD2.0 database. Protein–protein interaction (PPI) information was used to filtrate potentially false-positive hits. A “medium” threshold was chosen in GPS 5.0. The Gene Set Enrichment Analysis (GSEA) method was used to predict kinase activities, in which log-transformed phosphorylation levels (or ratio values) as a rank file and kinase-phosphorylation site regulations were formatted into a GMT format file in a sample (or a comparable group). Normalized enrichment scores (NES) were obtained from enrichment results and were regarded as kinase activity scores. Kinase was predicted as positive if the predominant change in substrates was an increase in phosphorylation and vice versa. For each comparable group, kinases predicted as having positive or negative activity and as having significantly differentially expressed phosphorylation sites were used to construct kinase–substrate regulatory network, according to the complicated regulatory relationships.

### Protein Extraction and Immunoblotting

The GCs were harvested and washed once in phosphate-buffered saline (PBS), then lysed on ice for 30 min with radioimmunoprecipitation assay (RIPA) buffer (CST, 9806), and supplemented with 1% (v/v) protease inhibitor Cocktail (HY-K0010) and 1% (v/v) phosphatase inhibitors (Cocktail I, HY-K0021; Cocktail II, HY-K0022; and Cocktail III, HY-K0023), which were purchased from MCE (Shanghai, China). Western blotting was performed as described previously (Wu et al., 2019; Yang et al., 2020a). Protein concentrations were determined using a BCA protein assay kit (TransGen Biotech, Beijing, China). Equal amounts of proteins (15–50 μg/lane) were separated by SDS-PAGE (12% acrylamide running gel) and transferred to a nitrocellulose membrane (BioTrace^TM^ NT; Pall Corp., FL, United States). The following antibodies were used in this experiment: beta catenin (ab32572; Abcam), inhibin alpha (ab81234; Abcam), HSD17B1 (ab134193; Abcam), MIF (ab227073; Abcam), caspase6 (ab185645; Abcam), laminA/C (MA3-1000; Thermo), and p-laminA/C-S22 (13448; CST, Shanghai, China). The antibodies were diluted to the recommended ratio with Beyotime (P0256; Shanghai, China) diluent. The Western blotting images were analyzed using ImageJ software (National Institutes of Health, Bethesda, MD, United States).

### Immunohistochemical and Immunofluorescence

Porcine follicles (3–5 mm in diameter) of different health statuses were fixed in 4% phosphate-buffered formaldehyde at 4°C for 7 days and then embedded in paraffin. Randomly selected sections (5 μm each) were used for subsequent staining. Immunohistochemical and immunofluorescence were performed as previously described (Wu et al., 2019; Yang et al., 2020a). MIF (1:500), laminA/C (1:100), and caspase6 (1:1,000) antibodies were used for staining. Immunohistochemical slides were examined under light microscopy (Leica DC200 digital camera; Leica, Germany). Immunofluorescence samples were examined using a confocal microscope (A1HD25; Nikon, Japan), and images were recorded.

### Detection of the MIF Concentration in Follicular Fluid and MIF mRNA Relative Expression in GCs

Follicular (3–5 mm in diameter) contents from H, SA, and A follicles were punctured by a hypodermic needle. Follicle fluid was obtained *via* centrifuging at 600 × *g* for 10 min. MIF concentration in the follicular fluid was detected using a MIF ELISA kit (JLC30201; Shanghai Jichun Industrial Co., Ltd., China). MIF mRNA relative expression in GCs was detected *via* Q-PCR. MIF primer: forward: ATCAGCCCGGACAGGATCTA, reverse: GCCGAGAGCAAAGGAGTCTT.

### Construction of Caspase6 DNA Mutant Vectors, Transfection, and Apoptotic Induction

The PCR-amplified full-length porcine caspase6 cDNA was constructed into pEGFP-N1 plasmid, forward primer: GAATTCatgagctcggcgctgga and reverse primer: GGATCCGCggattttggaaagaaatgc. The reading frame of caspase6 cDNA was connected with enhanced GFP to obtain a recombinant pEGFP-N1-caspase6 plasmid. The plasmid was propagated using Trans1-T1 (TransGen Biotech, Beijing, China) as the host strain and purified with a Plasmid MiniPrep Kit (EM111-01; TransGen Biotech), followed by sequencing to confirm the open reading frame (Sangon Biotech, Shanghai, China). Mutations of caspase6 Ser76 phosphorylation sites were performed by a Fast Mutagenesis System (FM111; TransGen Biotech), and primers for mutations were designed according to the manufacturer’s instructions. Based on the pEGFP-N1-caspase6 plasmid, porcine caspase6 Ser76 was mutated to alanine (caspase6-S76A), and caspase6 Ser76 was mutated to aspartic (caspase6-S76D). The primers used for caspase6S76A mutation are AccttaagcgcaggtttGcagatctaggatttgaag (forward) and tgCaaacctgcgcttaaggttgtctctgtcggca (reverse). The primers used for caspase6-S76D mutation are AccttaagcgcaggtttGACgatctaggatttgaag (forward) and GTCaaacctgcgcttaaggttgtctctgtcggca (reverse).

Lipofectamine^®^ 3000 reagent was used for plasmid transfection. The transfection protocol was according to the manufacturer’s instruction. Briefly, 1.5 μg of each plasmid DNA (pEGFP-N1, pEGFP-N1-caspase6, pEGFP-N1-caspase6-S76A, and pEGFP-N1-caspase6-S76D) and transfection reagent were added to a six-well plate cultured 293T cell. The control group was added with transfection reagent only. After transfection, the 293T cells were cultured for 16 h in humid air with 5% CO_2_ at 37°C for further apoptotic induction.

Apoptotic induction was performed as described previously (Saltiel, 2020). Briefly, transfected cells were treated with 30 μg/ml cycloheximide (CHX, SC0353; Beyotime, Shanghai, China) and 50 ng/ml tumor necrosis factor alpha (TNFα, #C600021; Sangon Biotech, Shanghai, China) for 12 h. Finally, the cells were harvested for Western blotting analysis. The protein levels of caspase6, cleaved caspase6, caspase3, and cleaved caspase3 were detected.

### Apoptosis Assay by Fluorescence-Activated Cell Sorter

293T cells were transfected and apoptosis-induced the same as described above. The cells were harvested and stained with Annexin V (PE) and 7-AAD (PerCP) in a binding buffer for 15 min according to the manufacturer’s instruction (abs50007; Absin, Shanghai, China). GFP positive cells were gated for apoptosis analysis by a fluorescence-activated cell sorter (FACS; Becton Dickinson, Franklin Lakes, NJ, United States), and the data were analyzed by FlowJo_V10 software.

### Statistical Analyses

Protein levels (gray values), MIF concentration, MIF mRNA relative expression levels, and cell apoptotic rates are presented as means ± SDs and were analyzed using a one-way ANOVA with SPSS 22 (IBM, SPSS, Chicago, IL, United States) for Windows. ANOVA followed by a *post hoc* Dunnett’s test was used to determine differences between different groups. Differences were considered statistically significant at *P* < 0.05.

## Results

### Overview of Proteomic and Phosphoproteomic Profiling of Porcine GCs During Atresia

**FIGURE 1:**
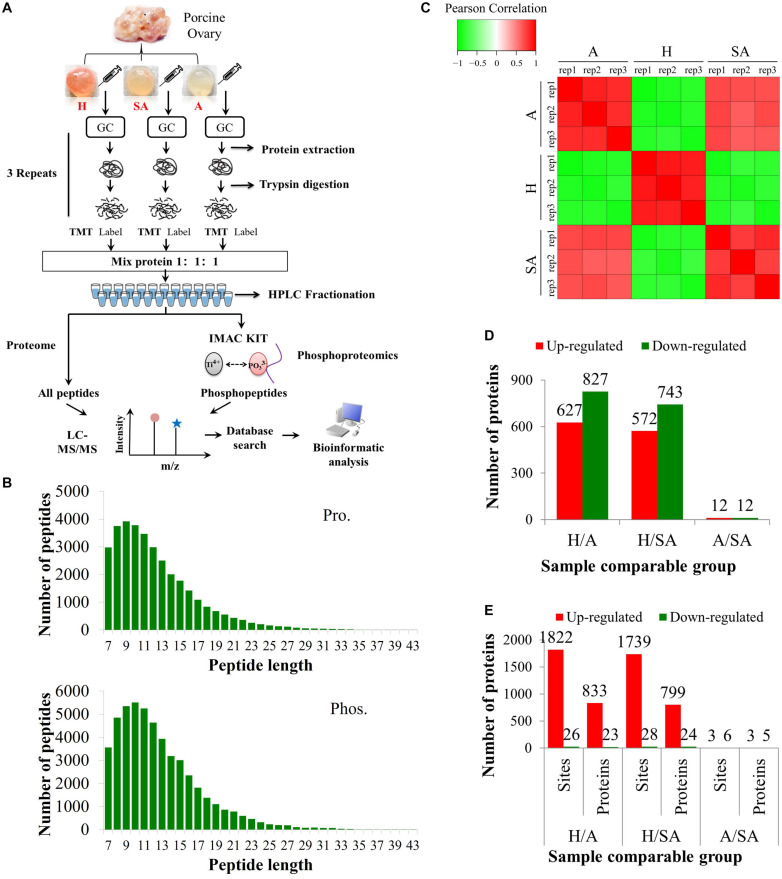
Overview of proteomic and phosphoproteomic analyses in granulosa cells obtained from ovaries with different follicular statuses. **(A)** Proteomic and phosphoproteomic analyses workflow in granulosa cells from healthy (H), slightly atretic (SA), and atretic (A) porcine follicle samples. **(B)** The distributions of raw MS/MS length of peptides and phosphopeptides quantified from proteomic and phosphoproteomic data, respectively. **(C)** Reproducibility analysis of the samples. **(D)** The amounts of DEPs in different comparable groups. **(E)** The amounts of the differentially quantified phosphosites and phosphoproteins in different comparable groups.

The proteomic and phosphoproteomic analyses workflow is illustrated in Figure 1A. The GC samples from H, SA, and A were lysed, digested, and labeled with different TMT tags, then pooled, and analyzed by LC/LC–MS/MS. Of the pool, 5% was used for proteome analysis, and the remaining 95% was subjected to phosphoproteome profiling. Altogether, 6,201 of the 7,104 identified proteins were quantified. In addition, 6,839 phosphorylation sites in 2,830 proteins were identified, among which 4,723 phosphorylation sites in 1,760 proteins were quantified. The length of most peptides varied between 8 and 20 in the proteomic and phosphoproteomic data, indicating that the sample preparation reached the standard (Figure 1B). Reproducibility analysis indicated high reproducibility between biological duplicate samples (Figure 1C). Relative quantitation of proteins was divided into two categories: a quantitative ratio over 1.5 was considered upregulation, whereas a quantitative ratio less than 0.67 was considered downregulation. The amount of DEPs in GC from different comparable groups is summarized in Figure 1D. The amount of the differentially quantified phosphorylation sites and differentially expressed phosphorylated proteins (DEPPs) from different comparable groups is summarized in Figure 1E.

### The Global Proteome Changes in GCs During Porcine Follicular Atresia

In this study, 6,201 of the 7,104 identified proteins were quantified. These data are presented in Supplementary Table 1, and the DEPs are presented in Supplementary Table 2. Among these, 24 (11 up, 13 down) proteins in H/SA and 50 (29 up, 21 down) proteins in H/A had a fold change (FC) higher than 5, which could be used as potential novel biomarkers to distinguish different health statuses of follicles, especially between H and SA. DEPs in H/SA follicles with an FC ≥ 5 are shown in Table 1.

**TABLE 1:** DEPs in H/SA follicles with an FC higher than 5.

Protein accession	Protein description	H/SA ratio	Regulated type	H/SA *P* value	Gene name	MW (kDa)
I3LV13	Uncharacterized protein	8.143854438	Up	0.00000952	PTMA	10.662
F1S5B3	Probable G-protein coupled receptor 125	7.915779673	Up	0.0249	−	49.116
K7GP96	Stathmin (fragment)	7.756051168	Up	0.0000627	STMN2	14.634
F2Z5V1	Uncharacterized protein	7.676805498	Up	0.00000775	TMA7	7.0662
P80928	Macrophage migration inhibitory factor	7.454297863	Up	0.0000285	MIF	12.451
F1RF36	G-protein coupled receptor 56	6.679439012	Up	0.00000929	ADGRG1	72.557
F1SPJ0	Partner of Y14 and mago	6.270759623	Up	0.00777	PYM1	22.893
P79304	Aromatase 3	6.049918674	Up	0.0017	CYP19A3	57.914
F1SNV1	Coiled-coil domain-containing protein	5.539910642	Up	0.00629	CCDC12	18.926
Q8WNW4	Beta-catenin	5.200604717	Up	0.00323	CTNNB1	85.51
Q6DUB7	Stathmin	5.044753902	Up	0.0234	STMN1	17.302
O02826	Beta-microseminoprotein	0.195162799	Down	0.000129	MSMB	12.246
F1S4V5	Actin-binding LIM protein 1	0.194795568	Down	0.0391	ABLIM1	89.92
F1RM86	ADAM DEC1	0.189244444	Down	0.0336	ADAMDEC1	51.441
A0A0C3SG01	Apolipoprotein A-I	0.183785283	Down	0.000167	APOA1	30.33
F1RHB4	C-X-C motif chemokine 14	0.179270868	Down	0.0274	CXCL14	11.744
F1RHI8	Cellular retinoic acid-binding protein 2	0.176317607	Down	0.0188	CRABP2	15.721
I3LLX8	Sulfotransferase	0.175809638	Down	0.0092	−	32.427
P32195	Protegrin-2	0.167075475	Down	0.00265	NPG2	16.478
F1SK59	Multidrug resistance-associated protein 1	0.165690185	Down	0.00427	ABCC1	147.09
I3LIS4	Macrosialin	0.16364486	Down	0.046	−	32.435
F1SB81	Plasminogen	0.159731479	Down	0.0000781	PLG	90.677
I3LP50	Vitronectin	0.151379668	Down	0.0000234	VTN	52.545
P48819	Vitronectin	0.119996481	Down	0.0000246	VTN	52.571

*H, healthy follicle; SA, slightly atresia follicle; MW, molecular weight.*

**FIGURE 2:**
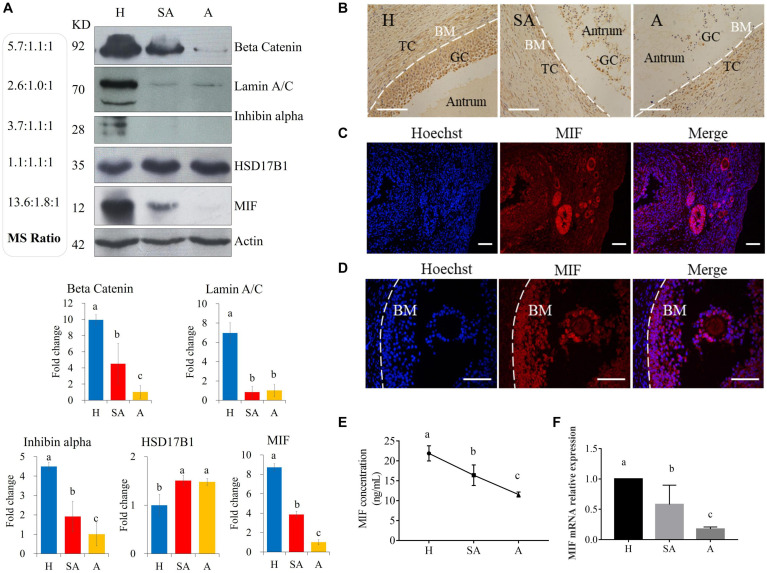
Western blotting validation of expression levels of target proteins found to be differentially expressed among H, SA, and A follicles by proteomics. **(A)** Validation of the proteome quantification. Protein levels of beta catenin, laminA/C, inhibin alpha, HSD17B1, and MIF were detected by Western blotting in granulosa cells from healthy, slightly atretic, and atretic porcine follicles. The histogram shows the quantitative analysis (mean ± SD) of the results of three independent Western blots. The bars are labeled with completely different letters (a, b, c) indicating significant difference, *P* < 0.05. **(B)** Immunohistochemical detection of MIF expression in pig healthy, slightly atretic, and atretic porcine follicles. **(C)** MIF is primarily expressed in granulosa cells of primordial follicles, primary follicles, secondary follicles, and small antrum follicles. **(D)** MIF is also expressed in cumulus cells. **(E)** MIF concentrations in follicular fluid from H, SA, and A follicles. **(F)** MIF mRNA levels in GCs from H, SA, and A follicles. H, healthy follicle; SA, slightly atretic follicle; A, atretic follicle; BM, basement membrane; GC, granulosa cell; TC, theca cell. Scale bar is 100 μm. The data are representative of at least three independent experiments.

Protein levels of β-catenin, laminA/C, inhibin alpha, HSD17B1, and MIF were validated *via* Western blotting, thus proving the reliability of the proteomic data (Figure 2A). MIF was 7.45 times higher in GCs from H than from SA follicles and 13.57 times higher in GCs from H than from A follicles and was confirmed by Western blotting and immunohistochemistry (Figures 2A,B). Immunofluorescence results showed that MIF was primarily expressed in GCs of primordial, primary, secondary, and small antrum follicles and was also expressed in cumulus cells (Figures 2C,D). MIF concentration in follicular fluid was highest in H compared with SA and A follicles (Figure 2E). In addition, the MIF mRNA expression level in GCs was the same as the protein profile in all three follicular categories (Figure 2F), which is consistent with a previous transcriptome study of porcine follicular atresia (Terenina et al., 2016). MIF can function as cytokine, hormone, and enzyme; however, its physiologic significance has yet to be elucidated (Nishihira and Jun, 2000; Calandra and Roger, 2003). In addition to inflammatory and immunologic functions, MIF plays a role in tumor cell growth, cell proliferation, wound repair, regulation of cytochrome c release, and inhibition of Bim-induced apoptosis (Hudson, 1999; Mitchell et al., 1999; Abe et al., 2000; Liu et al., 2008). Earlier studies in humans and mice revealed that MIF affects GC and theca cell proliferation, follicular growth, and ovulation (Shin-ichiro et al., 1999; Matsuura et al., 2002). Macrophages enter the ovarian follicle at the time of initiation of GC apoptosis and migrate as apoptosis processes (Yang et al., 2005). Macrophages can modulate follicle development by secreting epidermal growth factor (EGF) and other cytokines and suppress follicular cell apoptosis (Katabuchi et al., 1996). Macrophages may facilitate angiogenesis, express growth factors to enhance follicle growth, and secrete proteases to remodel the ECM (Liu et al., 2009). Taken together, this implies that MIF may play an important role in follicular development and could be used as a novel biomarker to distinguish different categories of follicle quality.

#### Functional Classification of DEPs

The amount of the DEPs in each GO term of level 2 was summed up according to the GO annotation information of identified proteins; these proteins can be found in Supplementary File 1. The results revealed that the DEPs in the cellular component were mainly concentrated in the cell, organelle, membrane, and macromolecular complex; the DEPs in the molecular function were mainly concentrated in binding, catalytic activity, transporter activity, structural molecule activity, molecular function regulator, and nucleic acid binding TF activity; the DEPs in the biological process were mainly concentrated in cellular process, metabolic process, single-organism process, biological regulation, response to stimulus, localization, and signaling; the DEPs in the subcellular location were mainly mapped in the nucleus, cytoplasm, mitochondria, extracellular membrane, and plasma membrane.

#### Functional Enrichment-Based Cluster Analysis

**FIGURE 3:**
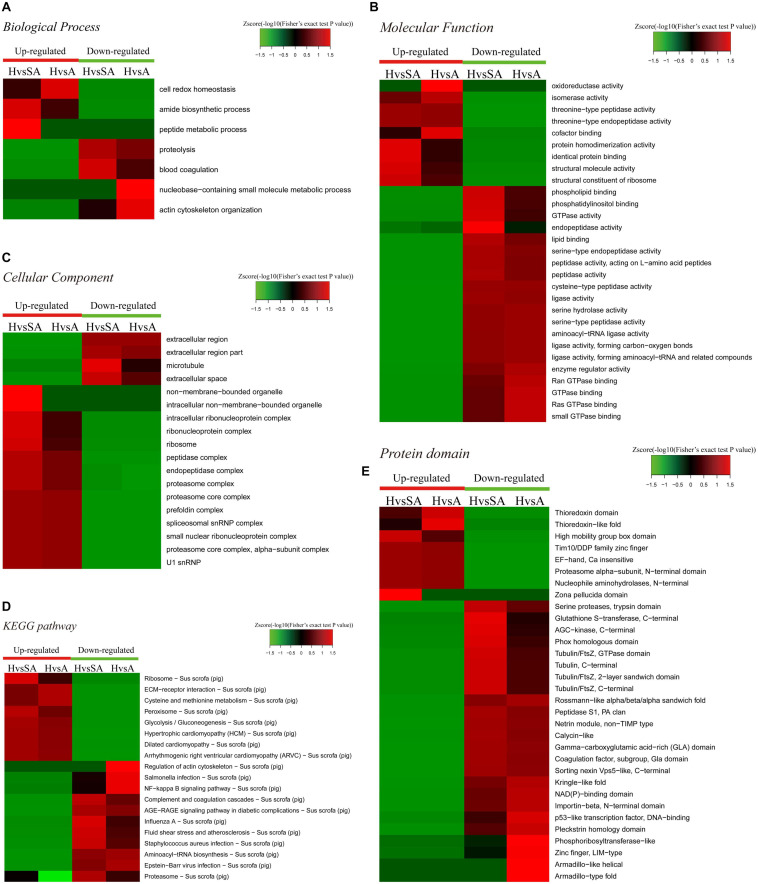
Heat map obtained from functional enrichment-based cluster analysis of the quantified proteomics datasets of different follicular statuses. **(A–C)** Heat maps obtained from GO enrichment-based cluster analysis. **(D,E)** Heat map obtained from enrichment-based cluster analysis of protein domains and KEGG pathways. H, healthy follicle; SA, slightly atretic follicle; A, atretic follicle.

A GO enrichment-based clustering analysis was performed to characterize the functions of the DEPs in GCs of different follicular statuses during atresia and included biological process, cellular component, KEGG pathway, molecular function, and protein domain (Figures 3A–C). In the biological process category (Figure 3A), amide biosynthetic process and peptide metabolic process were enriched in proteins that are higher in H than in SA; cell redox homeostasis was enriched among proteins that are higher in H than in A. In contrast, proteolysis and blood coagulation were enriched among proteins that are higher in SA than in H; nucleobase-containing small molecule metabolic process and actin cytoskeleton organization were enriched among proteins that are higher in A than in H. In the molecular function category (Figure 3B), protein homodimerization activity, identical protein binding, structural molecule activity, and structural constituent of ribosome were upregulated when follicles changed from H to SA. In addition, oxidoreductase activity, isomerase activity, and cofactor binding were upregulated when follicles changed from H to A. In contrast, phospholipid binding, phosphatidylinositol binding, GTPase activity, and endopeptidase activity were downregulated when follicles changed from H to SA; whereas, enzyme regulator activity, Ran GTPase binding, GTPase binding, Ras GTPase binding, and small GTPase binding were downregulated when follicles changed from H to A. In the cellular component-based clustering analysis (Figure 3C), non-membrane-bounded organelle, intracellular non-membrane-bounded organelle, intracellular ribonucleoprotein complex, ribonucleoprotein complex, ribosome, peptidase complex, endopeptidase complex, and proteasome complex were upregulated when follicles changed from H to SA. In contrast, extracellular region and extracellular region part were downregulated when follicles changed from H to SA and A. Microtubule and extracellular space were downregulated when follicles changed from H to SA. Enrichment-based clustering analyses were performed using the KEGG database to profile the cellular pathways (Figure 3D). The ribosome, peroxisome, and glycolysis/gluconeogenesis pathways were upregulated in H compared with SA. The ECM–receptor interaction as well as the cysteine and methionine metabolism pathways were upregulated when follicles changed from H to A. In contrast, complement and coagulation cascades, AGE–RAGE signaling pathway in diabetic complications, influenza A, fluid shear stress and atherosclerosis, *Staphylococcus aureus* infection, and proteasome were downregulated in H compared with SA. The regulation of actin cytoskeleton, salmonella infection, nuclear factor (NF)-kappa B signaling pathway, and aminoacyl-tRNA biosynthesis were downregulated when follicles changed from H to A. According to the analysis of the protein domain, high mobility group box domain and zona pellucida domain were upregulated when follicles changed from H to SA (Figure 3E). Thioredoxin-like fold was upregulated when follicles changed from H to A. In contrast, glutathione S-transferase-C-terminal, AGC-kinase-C-terminal, phox homologous domain, and tubulin related domains were downregulated when follicles changed from H to SA. Whereas, rossmann-like alpha/beta/alpha sandwich fold, kringle-like fold, NAD(P)-binding domain, importin-beta-N-terminal domain, p53-like TF–DNA-binding, pleckstrin homology domain, phosphoribosyl transferase-like, and zinc finger-related domains were downregulated when follicles changed from H to A.

Enrichment pathway images of DEPs in GCs of different follicular statuses can be found in Supplementary File 2.

### The Phosphoproteome Changes in GCs During Porcine Follicular Atresia

After IMAC enrichment and the LC–MS/MS analysis, 6,839 phosphorylation sites in 2,830 proteins were identified, and 4,723 phosphorylation sites in 1,760 proteins were quantified. These data are presented in Supplementary Table 3. The phosphoproteomic data were then normalized to the results of the global proteome analysis. The DEPPs can be found in Supplementary Table 4. There were 20 (up) and 39 (up) phosphorylation sites that had an FC ≥ 7 in the H/SA and H/A groups, respectively. In addition, 28 phosphorylation sites were downregulated in H/SA, and 26 phosphorylation sites were downregulated in H/A. The DEPPs in H/SA with an FC ≥ 7 are shown in Table 2.

**TABLE 2:** DEPPs in H/SA with an FC greater than 7.

Protein accession	Position	H/SA ratio	Regulated type	H/SA *P* value	Amino acid	Protein description (pig)	Gene name
F1RS45	1,548	9.433	Up	0.00252	Y	DNA topoisomerase 2	TOP2B
F1S620	109	9.429	Up	0.000000904	S	Putative RNA-binding protein 15	RBM15
F1S1M1	229	9.081	Up	0.0301	S	Synaptotagmin-like protein 4	LOC106507000
F1RL99	27	8.770	Up	0.000622	S	DNA ligase	LIG1
F1S5K7	573	8.645	Up	0.00856	T	Protein kinase C	PRKCE
F1RTQ8	1,321	8.282	Up	0.0312	S	F-actin-uncapping protein LRRC16A	CARMIL1
I3LIA3	410	8.226	Up	0.00373	S	Thyroid hormone receptor-associated protein 3	−
F1RUF9	354	8.038	Up	0.00253	S	Transcription factor SOX-4	SOX4
F1RFD8	1,710	8.002	Up	0.000112	S	E3 ubiquitin-protein ligase RBBP6	RBBP6
I3LFU8	3	7.833	Up	0.000678	S	Dedicator of cytokinesis protein 7	LOC100524027
F1SGB5	11	7.531	Up	0.0323	S	Endoplasmic reticulum–Golgi intermediate compartment protein 2	ERGIC2
F1SNU6	594	7.458	Up	0.00519	S	Histone-lysine N-methyltransferase SETD2	SETD2
F1S4U9	920	7.405	Up	0.0103	S	Echinoderm microtubule-associated protein-like 4	EML4
F1RJJ0	199	7.390	Up	0.00926	S	Serine/arginine-rich splicing factor 9	SRSF9
F1ST95	25	7.349	Up	0.00292	S	Oxysterol-binding protein	−
F1RVD4	804	7.288	Up	0.00101	S	Protein transport protein Sec31A	SEC31A
I3L7E5	730	7.157	Up	0.000445	S	DNA helicase	MCM3
F1RS45	1,515	7.120	Up	0.0000721	S	DNA topoisomerase 2	TOP2B
F1SK12	1,676	7.045	Up	0.00987	S	Microtubule-associated protein 1B	MAP1B
F1RS45	1,416	7.033	Up	0.00698	S	DNA topoisomerase 2	TOP2B

*H, healthy follicle; SA, slightly atresia follicle; S, serine; T, threonine; Y, tyrosine.*

#### Functional Classification of DEPPs

The amount of the DEPPs in each GO term of level 2 was summed up according to the GO annotation information of identified proteins; these proteins can be found in Supplementary File 3. Similar to the results in the proteome, the DEPPs in the cellular component were mainly concentrated in the cell, organelle, macromolecular complex, and membrane; the DEPPs in the molecular function were mainly concentrated in binding and catalytic activity; the DEPPs in the biological process were mainly concentrated in cellular process, metabolic process, single-organism process, biological regulation, response to stimulus, signaling, and localization.

**FIGURE 4:**
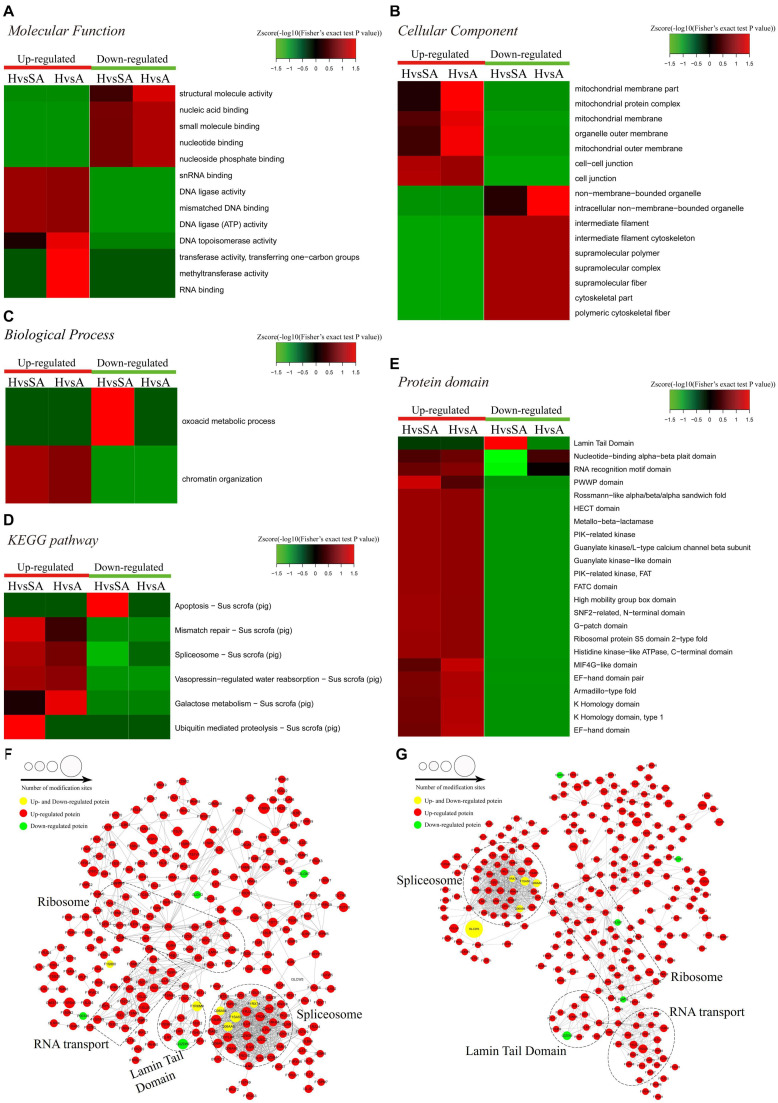
Heat map obtained from functional enrichment-based cluster analysis of quantified phosphoproteome during follicular atresia and protein–protein interaction networks of DEPs. **(A–C)** Heat maps obtained from GO enrichment-based cluster analysis. **(D,E)** Heat map obtained from enrichment-based cluster analysis of protein domains and KEGG pathways. Analyses of the protein–protein interaction networks for differentially expressed phosphorylated proteins between H and SA **(F)** and between H and A **(G)**. H, healthy follicle; SA, slightly atretic follicle; A, atretic follicle.

A functional enrichment-based clustering analysis was performed to characterize the functions of DEPPs in different comparable groups. In the molecular function category (Figure 4A), DNA topoisomerase activity, transferase activity-transferring one-carbon groups, methyltransferase activity, and RNA binding process were upregulated when follicles changed from H to A. In contrast, structural molecule activity, nucleic acid binding, small molecule binding, nucleotide binding, and nucleoside phosphate binding were downregulated when follicles changed from H to A. In the cellular component category (Figure 4B), DEPPs were mainly located in the mitochondrial membrane part, mitochondrial protein complex, mitochondrial membrane, mitochondrial outer membrane, organelle outer membrane, cell–cell junction, cell junction, non-membrane-bounded organelle, and intracellular non-membrane-bounded organelle. As shown in Figure 4C, the biological process analysis revealed that the chromatin organization process was upregulated, whereas the oxoacid metabolic process was downregulated when follicles changed from H to SA. Then, KEGG clustering was performed to characterize the alterations in signaling pathways during follicular atresia (Figure 4D). Results revealed that mismatch repair, galactose metabolism, spliceosome, vasopressin-regulated water reabsorption, and ubiquitin-mediated proteolysis pathways were upregulated when follicles changed from H to SA or A. Importantly, only the apoptosis pathway was found to be downregulated when follicles changed from H to SA. According to the analysis of the protein domain (Figure 4E), the PWWP domain and MIF4G-like domain were upregulated when follicles changed from H to SA or A. However, the Lamin tail domain was downregulated when follicles changed from H to SA, which is involved in the apoptosis pathway.

Protein–protein interaction networks were established based on the proteins whose phosphosites were regulated during follicular atresia. Molecular complex detection showed that these proteins were mainly divided into four categories in both the H vs. SA (Figure 4F) and H vs. A (Figure 4G) groups: spliceosome, RNA transport, Lamin tail domain, and ribosome. Moreover, 69 sequence motifs were predicted from all the identified phosphorylation sites with Motif-x (Supplementary Figure 1).

**FIGURE 5:**
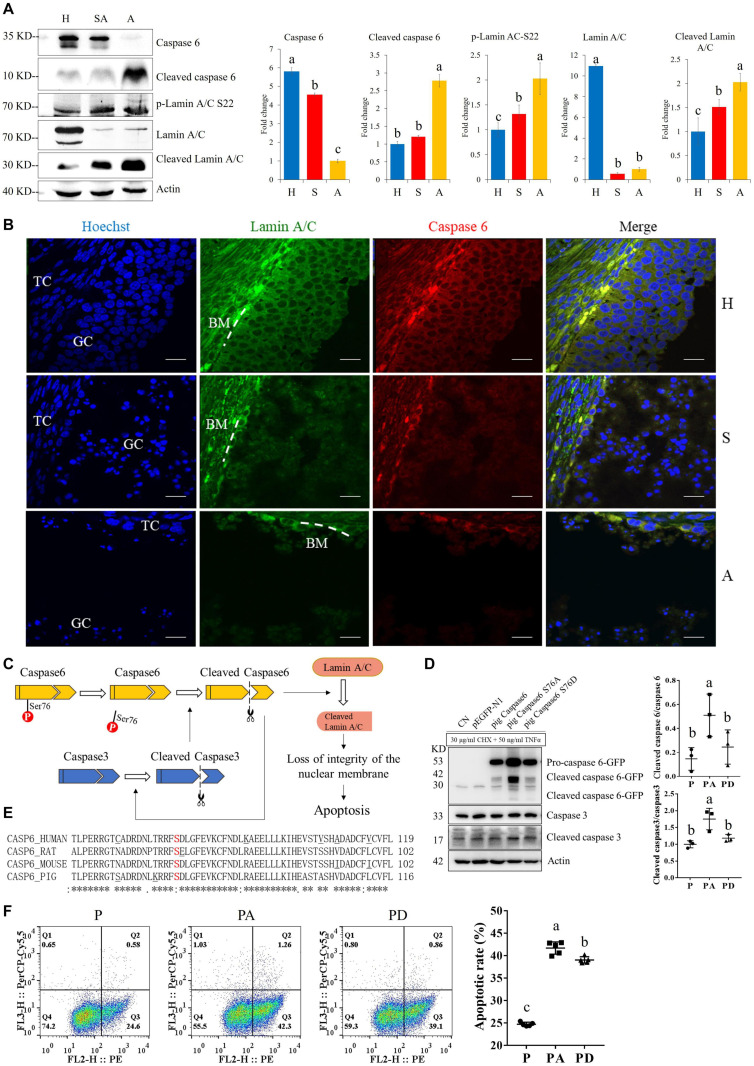
The expressions of caspase6, cleaved caspase6, p-laminA/C-S22, laminA/C, and cleaved laminA/C in GCs from healthy (H), slightly atretic (SA), and atretic (A) follicles, as well as the possible regulatory pathways of caspase6, caspase3, laminA/C, and apoptosis. **(A)** Protein levels of caspase6, cleaved caspase6, p-laminA/C-S22, laminA/C, and cleaved laminA/C in GCs from H, SA, and A follicles were detected by Western blotting. Quantitative analysis of these proteins is shown in histograms. The bars are labeled with completely different letters (a, b, c) indicating significant difference, *P* < 0.05. **(B)** The expression of laminA/C and caspase6 in GCs of follicles in different health states was detected by immunofluorescence. Nuclei were stained with Hoechst. Scale bar = 100 μm. TC, theca cell; GC, granulosa cell; BM, basement membrane. **(C)** Hypothetical model of the regulatory pathways of caspase6, caspase3, and laminA/C in GCs during follicular atresia. **(D)** Caspase6 S76A increased the ratios of caspase6 cleavage compared with the wild-type caspase6 and caspase6 S76D, after transfection and apoptosis induction. 293T cells were transfected with caspase6, caspase6 S76A, and caspase6 S76D plasmids for 16 h; the CN and p-EGFP-N1 are transfection control. Then, the cells were treated with 30 μg/ml CHX and 50 ng/ml TNFα for 12 h. The cell was harvested for Western blotting analysis. The ratios of cleaved caspase6/caspase6 and cleaved caspase3/caspase3 in the pig caspase6 (P), pig caspase6 S76A (PA), and pig caspase6 S76D (PD) groups were shown in the graph. The data are representative of three independent experiments. **(E)** Align the amino acid sequence near the Ser76 of caspase6 in human, rat, mouse, and pig. **(F)** Apoptotic rates of GFP positive cells after transfection with caspase6, caspase6 S76A, and caspase6 S76D plasmids. 293T cells were transfected and apoptosis-induced the same as described above and were analyzed by flow cytometry after Annexin V (PE) and 7-AAD (PerCP) staining. The data are representative of five independent experiments.

For the apoptosis pathway, 14 related proteins, including caspase3, were higher in GCs of SA than of H. The protein levels of laminA/C and lamin B were higher in GCs of H than of SA; however, phosphorylation levels of lamin A/C (S22 and S636) and lamin B1 (S308 and S534) were higher in GCs from SA than from H. Lamins, major structural proteins of the cell, are targeted for destruction early in apoptosis (Goldberg et al., 1999). Lamin A/C is cleaved to a small fragment (28 kDa), specifically by caspase6, but not by other caspases, during the induction of apoptosis (Orth et al., 1996; Goldberg et al., 1999). The PPI networks also showed direct interactions between caspase6 and lamin A/C (Figures 4F,G). Proteomic analysis showed no difference in the caspase6 protein levels in GCs from H, SA, and A. The phosphorylation level of caspase6 (S76) was higher in GCs from H than from SA. Phosphorylation occurs in all structural domains of caspases typically resulting in caspase inactivation by preventing the formation of the active, cleaved caspase (Duncan et al., 2011). Western blot results confirmed the protein levels of caspase6 and laminA/C, as well as the phosphorylation level of laminA/C at S22 in GC during follicular atresia, and showed that both cleaved caspase6 and cleaved laminA/C were higher in GC from A and SA than from H (Figure 5A). The expression patterns of laminA/C and caspase6 were also detected by immunofluorescence. Both laminA/C and caspase6 were expressed in the cytoplasm of GCs in H follicles. However, as the follicles underwent atresia, lamin A/C and caspase6 entered the nucleus, diffusely expressing in the apoptotic GCs, and their staining signals are also weaker (Figure 5B). A previous study showed that phosphomimetic substitution of S22 in lamin A/C resulted in an increase in laminA/C in the nucleoplasm (Kochin et al., 2014). However, a mutation of S22 to Ala on laminA significantly inhibits laminA disassembly in mitotic cells (Haas and Jost, 1994). The levels of phosphorylated S636 in laminA have been shown to increase two-fold in mitotic HeLa S3 cells (Chen et al., 2013). Activation of caspase3 can result in the activation of pro-caspase6, and activation of pro-caspase6 can also result in the activation of caspase3, resulting in a protease amplification cycle (Cohen, 1997; Allsopp et al., 2000). Collectively, we deduce that phosphorylation at S76 may block the activity of caspase6, therefore preventing lamin A/C from cleavage. Phosphorylation of laminA/C at S22 and S636 may contribute to laminA/C disassembly or cleavage, thus promoting apoptosis of GCs during follicular atresia (Figure 5C). Our further study showed that caspase6-S76A increased the cleaved caspase6/caspase6 and cleaved caspase3/caspase3 ratio compared with the caspase6 and caspase6 S76D group, after transfection and apoptosis induction (Figure 5D). Apoptotic rates of GFP positive cells after transfection with caspase6, caspase6 S76A, and caspase6 S76D plasmids were analyzed by flow cytometry. Results revealed that the apoptotic rate of cells transfected with caspase6 S76A was higher than that of cells transfected with caspase6 S76D and caspase6; the apoptotic rate of cells transfected with caspase6 S76D was higher than that of cells transfected with caspase6 (Figure 5F).

### TF Dynamics in GCs During Follicular Atresia

**FIGURE 6:**
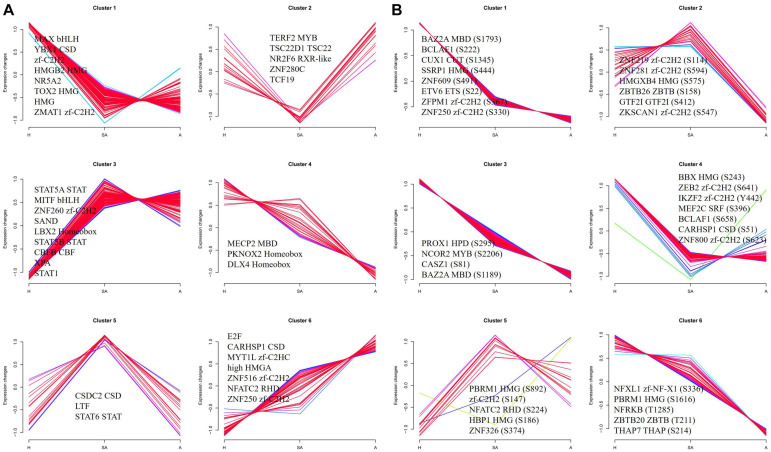
Clustering analysis of TF expression patterns and changes of phosphorylated TFs in GCs from three different follicular statuses. **(A)** Clustering analysis of TF expression patterns in proteome. **(B)** Clustering analysis of phosphorylated TF in phosphoproteome. Each line indicates the relative abundance of each protein. Selective proteins or phosphosites in each cluster are shown.

To investigate the dynamics of TFs along the steps of porcine follicular atresia, all 1,490 known porcine TFs from AnimalTFDB3 were analyzed (Hu et al., 2019). A total of 477 TFs were identified, and 359 TFs were quantified in the proteome. Cluster 1 comprised 127 TFs, which were upregulated in H compared with SA and A. Among these, MAX, YBX1, zf-C2H2, HMGB2, NR5A2, TOX2, HMG, and ZMAT1 had an FC ≥ 3, indicating that these TFs may play a critical role in follicular development. Cluster 3 consisted of 102 TFs, which were upregulated in SA and A compared with H. Among these, STAT5A, MITF, ZNF260, SAND, LBX2 Homeobox, STAT5B, CBFB, XPA, and STAT1 had an FC ≥ 2, suggesting that they are important in the initiation of atresia. Cluster 6 included 46 TFs, which were upregulated in A compared with SA and H. Among these, E2F, CARHSP1, MYT1L, HMGA, and ZNF516 had an FC ≥ 2, indicating that they are vital in the late stage of atresia (Figure 6A). In total, 173 of 307 identified TFs were quantified, and 572 of 850 identified sites were quantified in the phosphoproteome. Cluster 1, cluster 3, cluster 4, and cluster 6 consisted of 419, 142, 139, and 37 phosphorylation sites, respectively. All were upregulated in H compared with SA and A. Cluster 2 included 23 phosphorylation sites, which were highest in SA, second highest in H, and lowest in A. Cluster 5 consisted 14 phosphorylation sites, which were highest in SA, second highest in A, and lowest in H (Figure 6B). Taken together, some key TFs and phosphorylated TFs were found, which are involved in the normal development of follicles, initiation of follicle atresia, and eventually degeneration of follicles.

### Kinase Signaling Dynamics in GCs During Follicular Atresia

**FIGURE 7:**
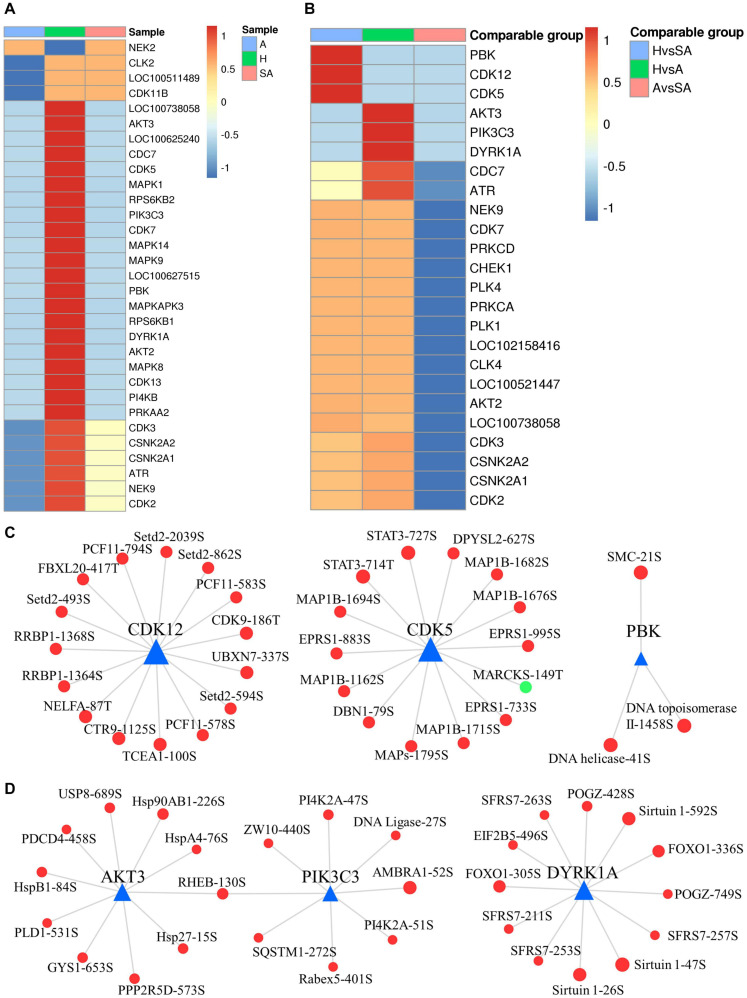
Dynamic changes of kinase activity and the key kinase–substrate regulatory network in GCs during follicular atresia. **(A)** Heat map of kinase activity scores in H, SA, and A. Columns are sample names, and rows are phosphokinases (rows are clustered and Z-score normalized). **(B)** Heat map of kinase activity matrix in comparable groups. The columns are the comparison group names, and the rows are phosphokinases (rows are clustered and Z-score normalized). **(C)** Map of key kinase–substrate regulatory networks in H/SA. **(D)** Map of key kinase–substrate regulatory networks in H/A.

A total of 2,762 regulatory relationships were predicted between 238 protein kinases, and 1,140 phosphorylated sites on 606 proteins were identified by phosphoproteomics (Supplementary File 4). The results of the predictive analysis of the kinase activity showed that 27 kinases in H were strongly activated, including several cell cycle kinases, including CK2 (CSNK2A1/2), CDC7, CDK5, CDK7, CDK13, and MAPKs (MAPK1, MAPK8, MAPK9, and MAPK14). However, NEK2 was blocked in H. In addition, nine kinases in A were predicted to be inactive, which also included several cell cycle kinases, CK2, CDK2, CDK3, CLK2, and CDK11B (Figure 7A). Kinase activity inferred from phosphoproteomic data indicated that PBK, CDK5, and CDK12 were upregulated in H/SA. In addition, AKT3, PIK3C3, DYRK1A, CDC7, and ATR were upregulated in the H/A group (Figure 7B). These upregulated kinases are mainly related to cell cycle, mitosis, and transcription. Kinase regulatory networks of PBK, CDK5, and CDK12 are shown in Figure 7C, and networks of AKT3, PIK3C3, DYRK1A are shown in Figure 7D. Of note, CDK12 was predicted to phosphorylate Setd2 at S493, S594, S862, and S2039. Setd2 is a specific methyltransferase for lysine-36 of histone H3, and methylation of this residue is associated with active chromatin (Yang et al., 2016). PIK3C3, an activated kinase in healthy follicles, was predicted to phosphorylate SQSTM1 at S272 and AMBRA1 at S52. Both of these proteins are autophagy regulators (Strappazzon et al., 2015); phosphorylation at these two sites inhibits autophagy (Cianfanelli et al., 2015; Sánchez-Martín and Komatsu, 2018). TF FOXO1 (S305 and S336) was phosphorylated by DYRK1A kinase and is involved in many biological processes including apoptosis, cell cycle arrest, stress resistance, glucose metabolism, cellular differentiation and development, and tumor suppression (Lu and Huang, 2011). Phosphorylation of FOXO proteins by DYRK1A has been reported to promote the export of these proteins to the cytoplasm (inactive), whereas phosphorylation by JNK can trigger the localization of FOXO proteins from the cytoplasm to the nucleus (Lu and Huang, 2011). The two phosphosites of FOXO1 found in this study are important for follicular development, prevent GCs from apoptosis, and may be valuable for developing compounds that disrupt the phosphorylation of FOXO1 by DYRK1A, which could serve as a potential choice for cancer-related drug design. DYRK1A was also predicted to phosphorylate Sirtuin 1, a histone deacetylase, at S26, S47, and S592. AKT3 was predicted to phosphorylate PDCD4 at S458, which may lead to ubiquitination and degradation of PDCD4, therefore inhibiting GC apoptosis and promoting translation (Schmid et al., 2008). The PDCD4 protein may be phosphorylated through the EGF-activated PI3K–AKT–mTOR–S6k1 signaling pathway and degraded in the proteasome system (Matsuhashi et al., 2019). AKT3 was also predicted to phosphorylate several HSP proteins, which play roles in stress resistance and actin organization.

## Discussion

Follicular atresia is a complicated process that limits the potential fertility of domestic animals. In this study, comprehensive proteomic and phosphoproteomic analyses were applied in GCs from different quality categories of follicles. Transcriptome analyses in GCs during follicular development and atresia have been performed in porcine (Bonnet et al., 2008; Terenina et al., 2016; Zhang et al., 2018a), sheep (Bonnet et al., 2011), bovine (Hatzirodos et al., 2014), and humans (Zhang et al., 2018b). Some key genes and pathways were found to be related to these processes. Proteomic analysis was also conducted in porcine follicular fluid during follicle development. The patterns of PPIs throughout follicle development, stage-specific proteins, and major pathways have been uncovered (Paes et al., 2019). However, the present study is the first proteomic and phosphoproteomic analyses of the process of mammalian follicular atresia. The large number of DEPs and DEPPs may provide valuable clues for future functional studies. The changes of TFs and kinases in GCs during atresia were also unveiled. In addition, dephosphorylation of caspase6 at Ser76 was found to be a new pathway of cell apoptosis and may lead to follicular atresia. An in-depth understanding of the mechanism of follicular atresia is helpful for the advancement of reproductive technologies, such as three-dimensional follicle culture, and developing artificial ovaries and may also provide some new ideas for fertility preservation of cancer patients.

In human-assisted reproduction and livestock production, it is critical to distinguish between healthy and atretic follicles and to evaluate oocyte quality. Morphological changes, apoptosis of GCs, detachment of the GC layer from the follicular BM, and an increased ratio of progesterone to estrogen (E_2_) can serve as powerful evidence of follicular atresia (Wang et al., 2012; Cao et al., 2015; Yang et al., 2020a). Evaluation of oocyte quality mainly depends on morphologic characteristics (Salazar et al., 2009). In this study, some DEPs (e.g., MIF, beta catenin) and DEPPs (e.g., S22 of laminA/C, S76 of caspase6) could serve as potential novel biomarkers to distinguish follicles of different health statuses and to evaluate oocyte quality.

Some novel phosphorylation sites of key proteins were found in the phosphoproteomic analysis, which may have important significance in the functional study of corresponding proteins and in clinical applications. For example, regulation of caspase6 activation is not clear. In this study, the phosphorylation level of S76 of caspase6 was found to be higher in GCs from H than from SA or A, which may be a key phosphosite to suppress caspase6 activation. Increasing evidence has shown that caspase6 is highly involved in axon degeneration and neurodegenerative diseases, such as Huntington’s disease and Alzheimer’s disease (Wang et al., 2015). Inhibiting the function of caspase6 may have therapeutic potential for various neurodegenerative disorders (Graham et al., 2011; Wang et al., 2015). Herein, we show that caspase6 Ser76A increased both the ratios of caspase6 and caspase3 cleavage and increased cell apoptotic rate. In addition, this phosphosite is located at a highly conserved position of caspase6 (Figure 5E). The Ser76 of caspase6 in porcine is conservative to Ser79 of caspase6 in humans. In addition, five phosphorylation sites (S30, S269, S274, S275, and T319) of HSD17B1 had higher levels of phosphorylation in GCs from H than from SA and A, which may contribute to the catalytic activity of HSD71B1 and promote E_2_ synthesis. This may explain the change in the ratio of progesterone to E_2_ during atresia. HSD17B1 is overexpressed in E_2_-dependent cancers, such as breast, endometrial, and ovarian cancers (Luu-The, 2001; Konings et al., 2018). Therefore, these five phosphosites may provide new insights into the treatment of these types of cancers. Another example is the change of phosphorylation level of the gap junction alpha-1 protein (Connexin43) at S328 and S262, which may affect gap junction formation and permeability during mitosis, thus affecting the transfer of materials between GCs (Cooper, 2002; Norris et al., 2008).

Differentially expressed proteins and DEPPs in the biological process were mainly concentrated in cellular process, metabolic process, single-organism process, biological regulation, response to stimulus, localization, and signaling. Results of the current study revealed that blood coagulation, proteolysis, and oxoacid metabolic process were elevated in SA, whereas peptide metabolic process, amide biosynthetic process, and chromatin organization process were damaged in SA. Nucleobase-containing small molecule metabolic process and actin cytoskeleton organization were mapped to be higher in A; however, cell redox homeostasis was destroyed in A. These most significantly affected biological processes may better explain the mechanisms of atresia.

Apoptosis, proteasome, and complement and coagulation cascades pathways were activated in SA, which may play critical roles in the initiation of atresia. Regulation of the actin cytoskeleton, NF-kappa B signaling pathway, and aminoacyl-tRNA biosynthesis pathway were increased in A, indicating that these pathways may contribute to the final degeneration of follicles. However, peroxisome, glycolysis/gluconeogenesis, vasopressin-regulated water reabsorption, mismatch repair, galactose metabolism, gap junction pathway, and steroid hormone biosynthesis pathways were upregulated in H, which may contribute to follicular development, whereas their downregulation may be the main reason of follicular atresia. Predicted pathways and the corresponding DEPs and DEPPs will be valuable for conducting further investigations and verification analysis of porcine follicular atresia. The proteomic and phosphoproteomic analyses in the current study found some of the same KEGG pathways as those found in the previous transcriptome: apoptosis, AGE–RAGE signaling pathway in diabetic complications, fluid shear stress and atherosclerosis, ECM–receptor interaction, and ribosome (Zhang et al., 2018a). Our analysis also revealed some new KEGG pathways that are involved in atresia, including peroxisome, cysteine and methionine metabolism, glycolysis/gluconeogenesis, ubiquitin-mediated proteolysis, spliceosome, mismatch repair, galactose metabolism, regulation of actin cytoskeleton, NF-κB signaling pathway, proteasome, and aminoacyl-tRNA biosynthesis.

In the regulation of actin cytoskeleton pathway, the levels of 23 proteins were higher in A than in H. Among these, cofilin-1 and integrin β2 were the most variable proteins. The actin cytoskeleton acquires a new conformation, a sphere-like structure that separates the apoptotic blebs from the main bulk of the cell and plays an important role in transmitting survival signals exerted by the BM, laminin, and growth factors that activate tyrosine kinase receptors (Amsterdam et al., 1998). Active cofilin can regulate the rearrangement of the actin cytoskeleton and required to initiate progesterone secretion by preovulatory GCs *via* the LHR-PKA signaling pathway (Karlsson et al., 2010). Integrins are the major cellular receptors mediating adhesion to the ECM, and integrin signaling regulates actin reorganization, cell proliferation, apoptosis, gene expression, differentiation, and cell migration (Monniaux et al., 2006). During folliculogenesis, the expression of integrin subunits in GCs varies significantly between animal species (Monniaux et al., 2006). It has been reported in sheep that α6 associates with the β1 integrin subunit to form the α6β1 laminin receptor in GCs of healthy follicles, and that the expressions of both subunits decrease with atresia (Le, 2002). Our data showed that integrin β2 in porcine GCs was about four times higher in A than in H, which may be important for GC apoptosis and follicular atresia.

In the NF-kappa B signaling pathway, the levels of nine proteins were higher in A than in H. Among these, Bcl10 was the most variable protein, which contains a caspase recruitment domain (CARD), and has been shown to induce apoptosis and to activate NF-κB (Bertin et al., 2001). TNF receptor-associated factor 6, which functions as a signal transducer in the NF-kappa B pathway that activates I kappa B kinase (IKK) in response to proinflammatory cytokines (Tsukamoto et al., 1999; Mochida et al., 2000), was also elevated in GCs from A follicles. Our earlier study showed that the inhibition of NF-κB increased autophagy *via* JNK signaling and promoted progesterone secretion in porcine GCs (Gao et al., 2016).

In the aminoacyl-tRNA biosynthesis pathway, the levels of 13 proteins, including seryl-tRNA synthetase, leucyl-tRNA synthetase, tyrosyl-tRNA synthetase, threonyl-tRNA synthetase, prolyl-tRNA synthetase, and methionyl-tRNA synthetase, were higher in A than in H, indicating that the aminoacyl-tRNA biosynthesis or metabolism was increased in GCs from A. ARSs play a vital role in protein synthesis by linking amino acids to their cognate transfer RNAs (tRNAs). Aminoacyl-tRNA has also been found to have functions in several other biosynthetic pathways, such as lipid and protein degradation (Francklyn and Mullen, 2019). The role of the aminoacyl-tRNA biosynthesis pathway in atretic follicles needs to be further studied.

In the ECM–receptor interaction, eight proteins were mapped to be higher in GCs from H than from SA. Among these, dystroglycan had the largest FC and was 4.27 times higher in GCs from H than from A. ECM plays an active and complex role in regulating the morphogenesis of cells that contact it, influencing their survival, migration, proliferation, and metabolic functions (Aharoni et al., 1997). In the mammary epithelium, loss of cell adhesion to the BM due to ECM proteolysis is believed to be sufficient to initiate apoptosis and involution of the gland (Taddei et al., 2003). Growth factors (such as the basic fibroblast growth factor) and/or adhesion molecules within the ECM are also related to cell survival, as well as proliferation (Guadagno et al., 1993; Ruoslahti and Reed, 1994). Increasing evidence suggests that various ovarian ECM components present in the follicular BM, around GCs, and in the follicular fluid are important regulators of GC activity (Monniaux et al., 2006). ECM can regulate the expression of StAR protein and P450scc in rat GCs, which stimulates progesterone production. Rat GCs cultured in ECM are protected from apoptosis and have a well-developed actin cytoskeleton that is extensively spread out (Aharoni et al., 1997). An earlier study suggested that the inner layers of GCs, distal to the BM, are more sensitive to apoptotic signals than cells in close contact with the BM where the antiapoptotic signal of the BM, and in particular of laminin, has a direct effect (Aharoni et al., 1997). In this study, laminin, collagen, integrin α6, and proteoglycan βDG were higher in GCs from H than from SA. Change of integrin α6 in porcine GCs was consistent with that in GCs of healthy follicles in sheep (Le, 2002).

In the ubiquitin-mediated proteolysis, 23 phosphosites of 13 proteins were higher in GCs from H than from SA. Phosphosites of S100, S313, Y1052, and S1053 in E3 ubiquitin-protein ligase TRIP12, as well as S841 in E_2_ ubiquitin-conjugating enzyme UBE2O, had an FC ≥ 3. Numerous cellular processes regulated by ubiquitin-mediated proteolysis include the cell cycle, DNA repair and transcription, protein quality control, and the immune response. Our recent study showed that melatonin may maintain follicular health by inducing BimEL ubiquitination to inhibit the apoptosis of GCs (Wang and Zeng, 2018).

Using the TF database, we found some key TFs and phosphorylated TFs, which are involved in the normal development of follicles, initiation of follicle atresia, and eventually degeneration of follicles. For example, BCLAF1 is a transcriptional repressor that interacts with several members of the BCL-2 family of proteins, and overexpression of this protein induces apoptosis (Kasof et al., 1999). BCLAF1 is an important NF-κB signaling transducer and C/EBPβ regulator in DNA damage-induced senescence (Shao et al., 2016). Our data indicate that the phosphorylation levels of BCLAF1 at S222 and S658 were higher in SA and A than in H, which may contribute to its ability to induce apoptosis.

Kinase analysis revealed that there are more active kinases in GCs from H than from SA and A. These predicted differential kinases are mainly involved in cell cycle, mitosis, and transcription. The regulation networks of these key kinases were established. TFs, such as STAT3 (T714 and S727), FOXO1 (S305 and S336), RUNX1 (T22 and S220), SP1 (S59), TCEA1 (S100), and UBTF (S389), were predicted to be regulated by these kinases, which may better explain the mechanism of follicular atresia. The inactivated kinases in GCs from A may contribute to the atresia process.

## Conclusion

In summary, this study provides a comprehensive profile of DEPs and DEPPs in healthy, slightly atretic, and atretic antral follicles in porcine. A large number of proteins and phosphosites were found to be different in the GCs from three different quality categories of follicles. Some potential novel biomarkers for follicular atresia, such as MIF, were found. Some novel phosphosites of key proteins were found in the phosphoproteomic analysis, which may have significance in functional studies of corresponding proteins and in clinical applications, such as caspase6 Ser76. Further analysis of the DEPs and DEPPs revealed several core proteins, key phosphosites, biological processes, KEGG pathways, TFs, and kinases that are involved in atresia. Further study showed that dephosphorylation of caspase6 at Ser76 can increase the ratio of cleaved caspase6/caspase6 and lead to cell apoptosis, a new potential mechanism of follicular atresia. The results in the current study could lead to a better understanding of the molecular regulation of ovarian follicular atresia.

## Data Availability Statement

The datasets presented in this study can be found in online repositories. The names of the repository/repositories and accession number(s) can be found in the article/Supplementary Material.

## Ethics Statement

The animal study was reviewed and approved by Ethical Committee of China Agricultural University.

## Author Contributions

FY, QL, YC, HY, and HW performed the research. FY and QL analyzed the data and wrote the manuscript. FY, QL, and SZ designed the research. All authors contributed to the article and approved the submitted version.

## Conflict of Interest

The authors declare that the research was conducted in the absence of any commercial or financial relationships that could be construed as a potential conflict of interest.

## Footnotes

1 http://bioinfo.life.hust.edu.cn/AnimalTFDB/

## Supplementary Material

Supplementary Figure 1 motif-x – Phosphoproteomic.

Supplementary Table 1 MS_identified_information-Proteomic.

Supplementary Table 2 Differentially expressed statistics-Proteomic.

Supplementary Table 3 MS_identified_information-Phosphoproteomic.

Supplementary Table 4 Differentially expressed statistics-Phosphoproteomic.

Supplementary File 1 Functional_classification-Proteomic.

Supplementary File 2 Enrichment_pathway_image-Proteomic.

Supplementary File 3 Functional_classification-Phosphoproteomic.

Supplementary File 4 Kinase analysis.
