# Supplementary material for: Integrative Proteomic and Phosphoproteomic Analyses of Granulosa Cells During Follicular Atresia in Porcine
Source: Front Cell Dev Biol. 2021 Jan 15;8:624985. doi: 10.3389/fcell.2020.624985 (PMC7843964; doi:10.3389/fcell.2020.624985)
Supplement: Supplementary Figure 1 — motif-x – Phosphoproteomic. [file Data_Sheet_1.ZIP › Supplemental Data/Fig S1--motif-x--Phosphoproteomic.pdf]

Motif Logo	Motif	Motif Score	Foreground		Background		Fold Increase
			Matches	Size	Matches	Size	
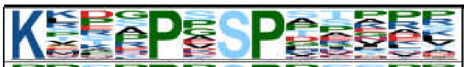	K...P.SP.....	38.15	42	5709	218	733195	24.74
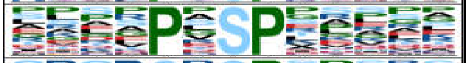	.....P.SP.....	32	483	5667	6078	732977	10.28
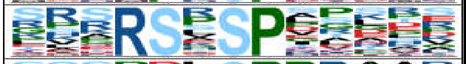	...RS.SP.....	39.51	109	5184	551	726899	27.74
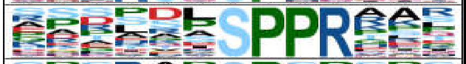	......SPPR...	41.54	55	5075	280	726348	28.11
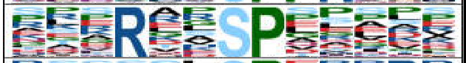	...R..SP.....	32	252	5020	2472	726068	14.74
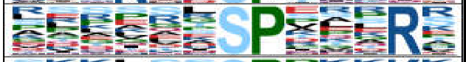	......SP...R.	32	215	4768	2662	723596	12.26
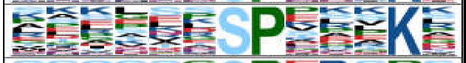	......SP...K.	32	186	4553	2147	720934	13.72
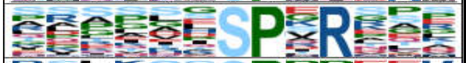	......SP.R...	32	158	4367	1879	718787	13.84
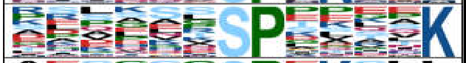	......SP....K	31.18	133	4209	1681	716908	13.48
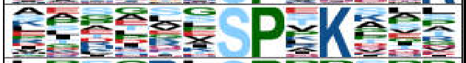	......SP.K...	29.25	118	4076	1591	715227	13.01
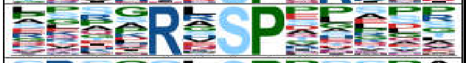	....R.SP.....	27.82	135	3958	2138	713636	11.38
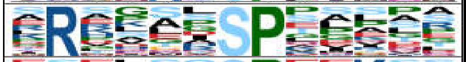	.R....SP.....	27.91	112	3823	1745	711498	11.95
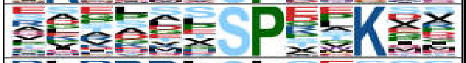	......SP..K..	25.94	82	3711	1287	709753	12.19
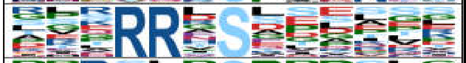	...RR.S......	32	196	3629	3194	708466	11.98
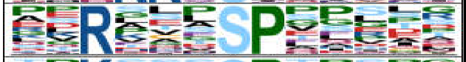	..R...SP.....	23.64	80	3433	1478	705272	11.12
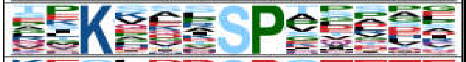	..K...SP.....	23.86	68	3353	1221	703794	11.69
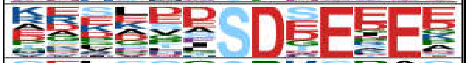	......SD.E.E.	40.06	55	3285	436	702573	26.98
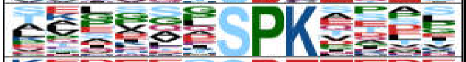	......SPK....	24.04	57	3230	981	702137	12.63
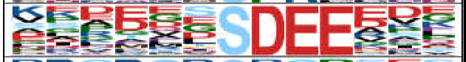	......SDEE...	43.3	39	3173	365	701156	23.61
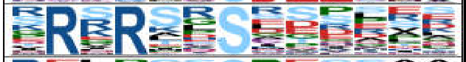	.R.R..S......	32	139	3134	3269	700791	9.51
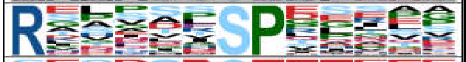	R.....SP.....	24	66	2995	1291	697522	11.91
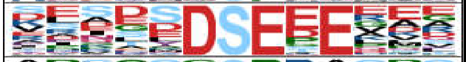	.....DSE.E...	42.23	40	2929	445	696231	21.37
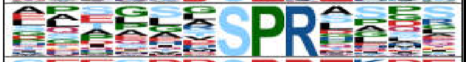	......SPR....	22.7	59	2889	1274	695786	11.15
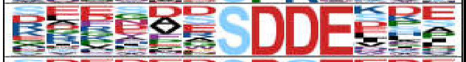	......SDDE...	38.36	27	2830	244	694512	27.16
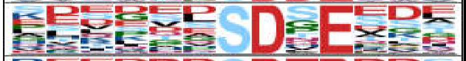	......SD.E...	32	76	2803	2412	694268	7.8
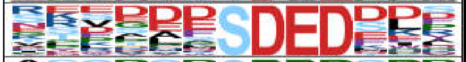	......SDED...	40.86	30	2727	285	691856	26.71
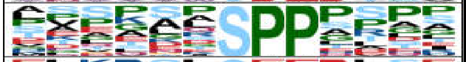	......SPP....	23.18	87	2697	2279	691571	9.79
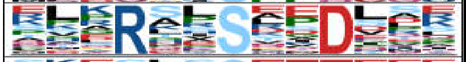	...R..S..D...	30.88	69	2610	1599	689292	11.4
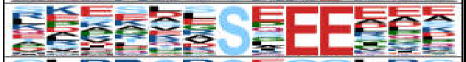	......S.EE...	28.81	110	2541	5115	687693	5.82
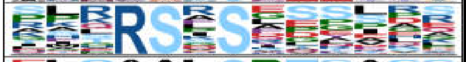	...RS.S......	32	80	2431	3638	682578	6.17
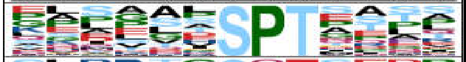	......SPT....	23.9	59	2351	1401	678940	12.16
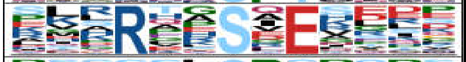	...R..S.E....	25.15	45	2292	1830	677539	7.27
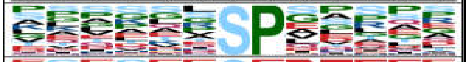	......SP.....	16	337	2247	19312	675709	5.25
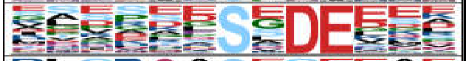	......S.DE...	32	69	1910	2280	656397	10.4
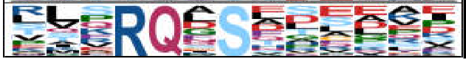	...RQ.S......	24.2	39	1841	1305	654117	10.62

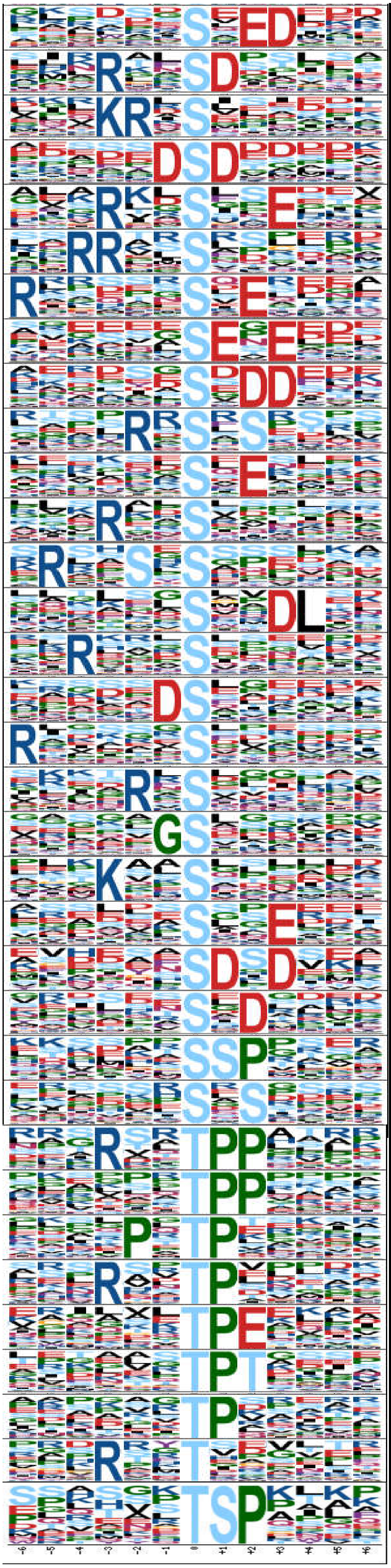

......S.ED...	27.06	55	1802	2794	652812	7.13
...R..SD.....	22.5	29	1747	1043	650018	10.35
...KR.S......	29.49	49	1718	2131	648975	8.69
.....DSD.....	32	50	1669	1857	646844	10.44
...R..S..E...	23.57	29	1619	1595	644987	7.24
..RR..S......	22.96	28	1590	1620	643392	6.99
R.....S.E....	22.69	29	1562	1826	641772	6.53
......SE.E...	22.82	37	1533	2929	639946	5.27
......S.DD...	29.26	42	1496	1792	637017	9.98
....R.S.S....	23.05	42	1454	3258	635225	5.63
......S.E....	16	159	1412	30848	631967	2.31
...R..S......	16	149	1253	20340	601119	3.51
.R..S.S......	22.51	38	1104	3713	580779	5.38
......S..DL..	22.7	36	1066	2942	577066	6.62
..R...S......	15.95	123	1030	30479	574124	2.25
.....DS......	13.08	97	907	25397	543645	2.29
R.....S......	12.85	96	810	27029	518248	2.27
....R.S......	9.92	69	714	20268	491219	2.34
.....GS......	10.27	97	645	35179	470951	2.01
...K..S......	8.63	69	548	25407	435772	2.16
......S..E...	7.23	57	479	22623	410365	2.16
......SD.D...	14.06	14	422	1064	387742	12.09
......S.D....	7.52	47	408	18387	386678	2.42
......SSP....	12.6	23	361	3961	368291	5.92
......S.S....	6.11	72	338	43491	364330	1.78
...R..TPP....	38.33	26	651	167	459774	109.96
......TPP....	32	113	625	2512	459607	33.08
....P.TP.....	28.86	73	512	3179	457095	20.5
...R..TP.....	27.53	39	439	1432	453916	28.16
......TPE....	22.02	33	400	2088	452484	17.88
......TPT....	22.69	26	367	1528	450396	20.88
......TP.....	16	95	341	20538	448868	6.09
...R..T......	10.61	42	246	22708	428330	3.22
......TSP....	19.01	21	204	2883	405622	14.48
